# DFT-guided synthesis of N, B dual-doped porous carbon from saccharina japonica for enhanced oxygen reduction catalysis

**DOI:** 10.3389/fchem.2024.1478560

**Published:** 2024-11-06

**Authors:** Junjie Zhang, Chao Wu, Jilong Wang, Maosong Xia, Shixin Li, Long Liu, Wuguo Wei, Xing Peng

**Affiliations:** College of Aeronautical Engineering, Civil Aviation Flight University of China, Chengdu, China

**Keywords:** ORR, DFT, N and B dual-doping carbon, fuel cell, biomass

## Abstract

**Introduction:**

The oxygen reduction reaction (ORR) is a crucial determinant of the energy transformation capacity of fuel cells. This study investigates the performance of N and B dual-doped carbon in ORR.

**Methods:**

Six models using density functional theory (DFT) are developed to compare the performance of different doping strategies. A highly efficient dual-doped carbon ORR catalyst (S-850-1) is synthesized from *Saccharina japonica*, containing 4.54 at% N and 1.05 at% B atom.

**Results:**

Electrochemical analysis reveals that S-850-1 significantly outperforms the nitrogen mono-doped carbon S-850, exhibiting a higher half-wave potential of 0.861 V and a greater limited current density of −5.60 mA cm⁻^2^, compared to S-850’s 0.838 V and −5.24 mA cm⁻^2^. Furthermore, S-850-1 surpasses the performance of 20% Pt/C, demonstrating enhanced durability and exceptional resistance to CO and methanol. The 1.40 V open circuit voltage produced by S-850-1 when integrated into a Zn-air battery can power an LED light.

**Discussion:**

Both theoretical and practical evaluations validate the excellent ORR performance of nitrogen and boron dual-doped carbon, as evidenced by the agreement between the electrochemical results and DFT calculations. This work not only extends the range of ORR catalysts derived from biomass but also provides guidance on creating and producing affordable, effective catalysts that utilize natural resources.

## 1 Introduction

Fuel cells (FCs) are particularly important due to their potential for integration into the hydrogen cycle (Hren et al., 2021; Mao et al., 2022). FCs are an innovative technology that efficiently and quietly converts chemical energy into electricity, offering significant ecological benefits. Their vast potential spans applications in small electronic devices, civil aviation, and aerospace equipment (Xiao et al., 2021). The oxygen reduction process is the main element influencing FCs’ energy conversion efficiency (ORR). Platinum (Pt), a noble metal, is considered the best catalyst for ORR (Su et al., 2021). The performance of the ORR catalyst is crucial for the efficiency of FCs (Arif et al., 2024). However, the limited durability, high-cost, and vulnerability to CO and methanol poisoning of Pt, along with its restricted global supply, hinder its economic feasibility (Liu et al., 2024). As a result, many researchers are investigating substitute catalysts, including low precious metal alloy catalysts, heteroatom-doped carbon catalysts, and transition metal-based catalysts (Bhoyate et al., 2023). Heteroatom doping carbon ORR catalysts have attracted a lot of attention because of their improved activity, extended stability, and outstanding resistance to methanol and CO (Gu et al., 2018).

Common heteroatoms used include boron (B), sulfur (S), phosphorus (P), and nitrogen (N) (Choi et al., 2013; Ferrero et al., 2016; Choi et al., 2012; Yang et al., 2012; Zhao et al., 2017; Cai et al., 2019). These heteroatoms may alter the electron distribution in nearby carbon atoms, improving oxygen molecule adsorption, reduction, and desorption, due to their different electronegativities from carbon (Zheng et al., 2012; Higgins et al., 2014). In contrast, biomass-derived carbon catalysts doped with heteroatoms offer several inherent advantages, including an abundant supply of natural heteroatoms, lower synthesis costs, and environmental sustainability (Borghei et al., 2018; Wang et al., 2020). Research indicates that carbon materials from biomass sources such as water hyacinth, soybean, okara, bamboo fungus, and grape skin are often mono-nitrogen doped (Liu et al., 2015; Guo et al., 2015; Gao et al., 2014a; Gao et al., 2014b; Chen et al., 2014; Alatalo et al., 2016; Zhao et al., 2018; Zhang et al., 2023a; Huang et al., 2020). Few studies have investigated carbon derived from biomass with dual heteroatom doping, with most research focusing on N and S dual doping in materials such as feathers, seaweed, chrysanthemum, and honeysuckle (Liu et al., 2014; Xu et al., 2017; Gao et al., 2015a; Gao et al., 2015b). Compared to single heteroatom doping, introducing multiple types of heteroatoms can further increase the asymmetry of carbon atoms’ spin and charge density, potentially enhancing ORR performance (Huang et al., 2020). Additionally, external boron doping in N-doping carbon has been demonstrated to further improve ORR performance (Wei et al., 2021). For instance, the current density and half-wave potential of B doping N doped carbon have been reported to increase from −0.23 to −0.21 V and from −5.26 to −5.64 mA cm^−2^, respectively (Huang et al., 2017; Zeng et al., 2020). Few studies have focused on biomass-derived dual heteroatom-doped carbon catalysts, with most previous research emphasizing the synthesis of N and B dual-doping carbon using chemical precursors (Lee et al., 2016; Li et al., 2022). Developing a dual-doped carbon ORR catalyst from biomass could present a strong candidate for commercial applications, offering distinct advantages: high ORR activity, cheap, sustainability, and environmental friendliness.

The brown algae saccharina japonica (phylum phaeophyta) is rich in protein, with content ranging from 6.8% to 10.3%, and serves as both a food source and a valuable source of heteroatoms (Xu et al., 2014; Su et al., 2022). Proteins, which are predominantly composed of nitrogen-rich amino acids, are the primary contributors of nitrogen. Boron is sourced from NaBH₄. This study examines the efficiency of N and B dual-doping carbon using DFT calculations. Saccharina japonica is rich in N element, and NaBH₄ serves as a source of both B and N atoms. Through high-temperature pyrolysis, dual-doped carbon materials containing N (4.54 at%) and B (1.05 at%) are produced. S-850-1, the synthesized sample, has a better limited current density (−5.60 mA cm^2^) and half-wave potential (0.861 V) than N-doping carbon (S-850: 0.838 V and −5.24 mA cm^2^). S-850-1 shows promise as ORR catalyst in FCs, considering the abundance of saccharina japonica worldwide and the rarity of N, B dual-doping carbon generated from biomass.

## 2 Experimental

### 2.1 Materials

Saccharina japonica is cleaned with water, and then baked to dryness. After being dried and powdered into a fine powder (5 g), 10 g ZnCl_2_ and 500 mL water are combined, and the above mixture are constantly agitated for 48 h. Above mixture is baked in an oven (80°C) until a fully dry colloid form. This colloid undergoes pyrolysis into a quartz at 850°C for 2 h. Above carbon material is combined with NaBH_4_ at a 1:1 mass ratio. The heated material is washed repeatedly with 2 M HCl solution and water until it reaches a neutral pH. The final product is labeled S-850-1. The sample, prepared using the same method as described above without the addition of ZnCl_2_, is designated as SN-850-1. The pyrolysis temperature of the samples is increased from 850°C to 1,000°C, with holding times of 2 and 3 h, respectively, denoted as S-1000-2 and S-1000-3. S-1000-2 is mixed with NaBH₄ in a 1:1 ratio, with the pyrolysis temperature set at 850°C and held for 2 h, resulting in the sample being named S-850-B.

### 2.2 DFT calculations

The population analysis, electron density difference, and adsorption free energy of O*, OH*, and OOH* (descriptors) are calculated using VASP software. The exchange-correlation functional is set to GGA-PBE. The energy cutoff for the plane-wave basis set is 500 eV, and the projector augmented wave method is used for pseudopotentials. A solvation model is applied using implicit solvation, and the dielectric constant for water (H₂O) is set to 78.5. The slab model is based on a graphene structure with a (4 × 4 × 3) supercell, with the bottom two layers of the slab fixed during relaxation. A vacuum layer of 20.0 Å is included, and the cleave plane is oriented along the (002) direction.

### 2.3 Characterization

D/MAX-Ultima + diffractometer with Co. Kα radiation is used for XRD. Using a WBL-810 apparatus, N_2_ adsorption-desorption tests are carried out at 77 K. The microstructural morphology of the produced samples is examined with a Supra-55 sapphire fitted FE-SEM.

### 2.4 Electrochemical measurements

The electrochemical studies are done using a Corrtest CS310 M electrochemical station. Following a combination of 10 mg sample, 5 mL isopropanol, and 20 µL 5% Nafion, the mixture underwent a 20 min ultrasonography treatment. Experiments using LSV (linear sweep voltammetry) and CV (cyclic voltammetry) are conducted at scan speeds of 10 and 20 mV s^−1^, respectively. At rotating speeds from 400 to 2,500 rpm, LSV curves are recorded in alkaline electrolyte.

To get the transferred electron number (N), the Koutechy-Levich (K-L) curve’s slope is calculated using Equations 1, 2.

1J=1JL+1JK=1Bω12+1JK (1)

B=0.2nFC0D23ν−16 (2)

The current density measured during the electrochemical experiments is denoted as *J*, while *J*_*k*_ represents the kinetic current density and *J*_*L*_ corresponds to the limited current density. n is the representation of the transmitted electron number. B represents the Levich slope, and the electrode rotating rate is indicated by ω. The Faraday constant, denoted as F. The diffusion coefficient of O_2_ is represented as D, and the bulk concentration of O_2_ is denoted as C. v is the kinetic viscosity representation.

## 3 Results and discussions

### 3.1 Model design

**SCHEME 1:**
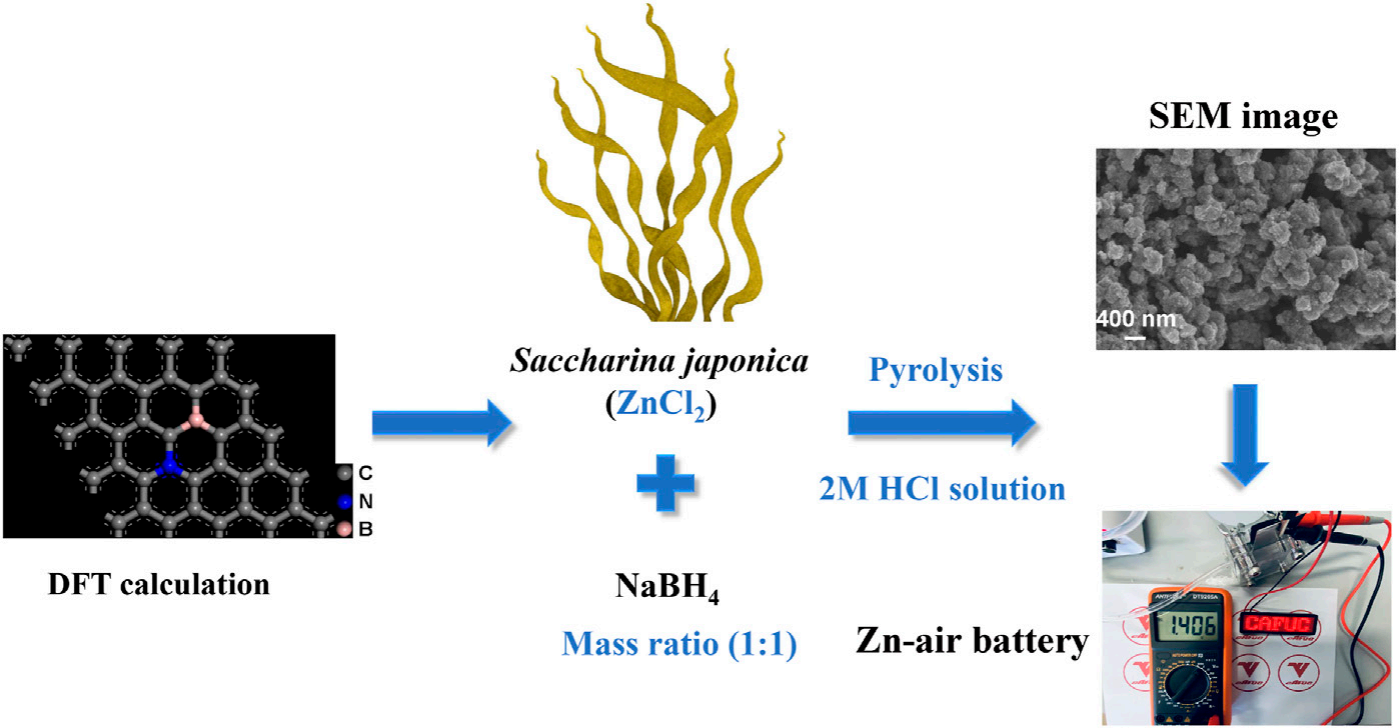
The synthesis process of N and B dual-doping carbon ORR catalyst derived from saccharina japonica.

This study uses DFT calculations to confirm that the ORR activity of the N, B dual-doping carbon model is higher than that of the N or B mono-doping carbon models. Saccharina japonica and NaBH_4_ are employed as sources of N and B, respectively. High-temperature pyrolysis and washing with 2 M HCl solution are then applied to produce carbon materials doped with N (4.54 at%) and B (1.05 at%) as shown in Scheme 1.

**FIGURE 1:**
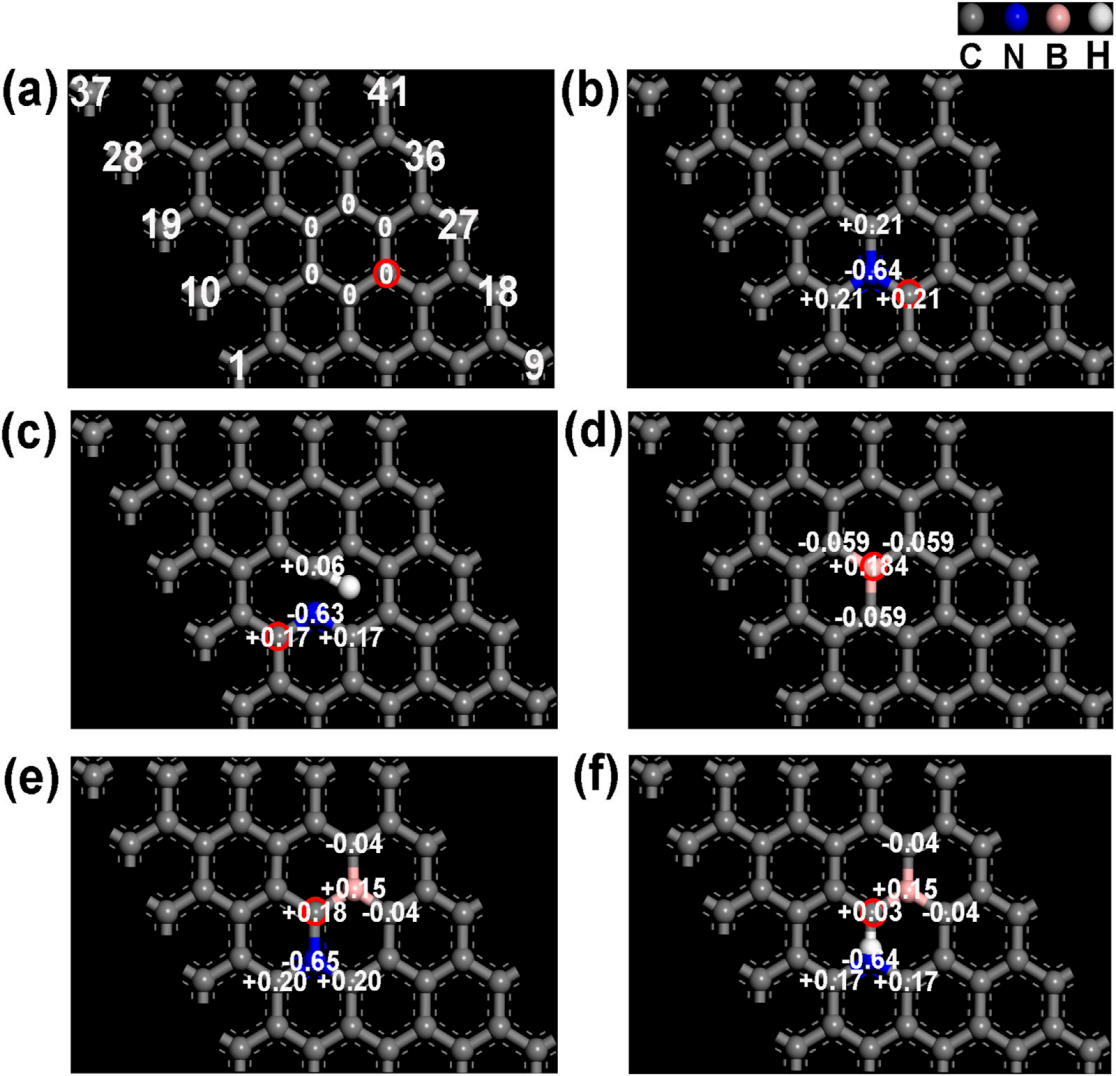
**(A)** Graphene, **(B)** graphitic-N doped carbon, **(C)** pyridinic-N doped carbon, **(D)** B doped carbon, **(E)** graphitic-N, B dual doped carbon, and **(F)** pyridinic-N, B dual doped carbon’s charge distribution images.

**FIGURE 2:**
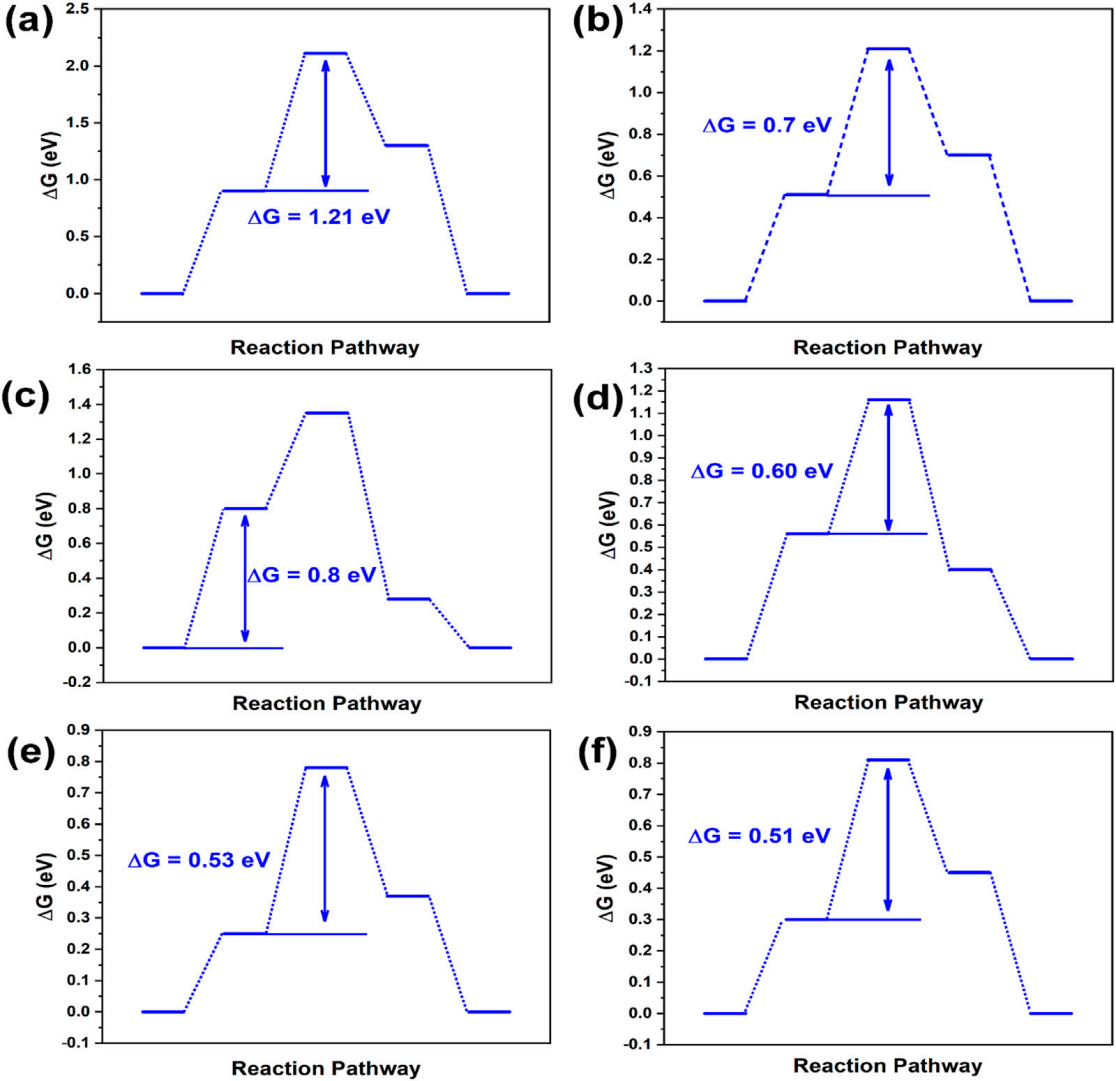
Energy step images of **(A)** graphene, **(B)** graphitic-N doped carbon, **(C)** pyridinic-N doped carbon, **(D)** B doped carbon, **(E)** graphitic-N, B dual doped carbon, and **(F)** pyridinic-N, B dual doped carbon.

Reports commonly identify graphitic and pyridinic nitrogen as the most effective active sites for ORR (Guo et al., 2020; Liang and Yuan, 2024). Figures 1, 2 depict reaction energy barriers and the charge distribution for models of graphene, pyridinic-N, graphitic-N, and B-doping carbon. The O*, OH*, and OOH* species exhibit favorable adsorption and desorption energies, as shown by their adsorption at the reaction sites marked by red circles in these models. The graphene model demonstrates a homogeneous charge distribution, as seen in Figures 1A, 2A, leading to a substantial energy barrier for the reaction (1.21 eV in step 2) and limited adsorption capacity. As shown in Figure 1B, graphitic-N (N_13_) is bonded to three carbon atoms (C_12_, C_14_, C_23_), forming three C-N bonds. The electronegativity of N atom (3.04) is higher than that of C atom (2.55), causing the N atom to attract the valence electrons of the C atoms. As a result, N atom accumulates electrons, gaining a charge of −0.64 e, while the three surrounding C atoms (C_12_, C_14_, C_23_) lose valence electrons, each acquiring a charge of +0.21 e. The reaction energy barrier of the graphitic-N doped carbon model is reduced to 0.70 eV in step 2 (Figure 2B), attributed to the uneven distribution of the electron cloud.

In Figure 1C, the pyridinic-N atom forms C-N bonds with two carbon atoms (C_12_ and C_14_). Due to the presence of a lone pair of electrons, pyridinic-N exhibits a weaker electron-attracting ability compared to graphitic-N. Consequently, C_12_ and C_14_ carry a charge of +0.17 e, while C_23_ exhibits a charge of +0.06 e. Due to the uneven electron cloud distribution, the reaction energy barrier of pyridinic-N doped carbon is reduced to 0.80 eV (step 1) in Figure 2C.

Electronegativity of B atom (2.04) is lower than that of C atom, causing its electrons to be attracted by the surrounding C atoms (C_13_, C_22_, and C_24_). B atom carries a charge of +0.184 e, while the neighboring C atoms (C_13_, C_22_, and C_24_) carry a charge of −0.059 e in Figure 1D. The reaction energy barrier of B-doped carbon is further reduced to 0.60 eV (step 2) in Figure 2D. In Figure 1E, the dual doped graphitic-N and B atoms further disrupts the electron cloud distribution. The electron density of graphitic-N atom increases to −0.65 e, while the electron density of B atom decreases to +0.15 e. The surrounding carbon atoms, C_12_ and C_14_, carry a charge of +0.20 e, C_23_ carries +0.18 e, while C_25_ and C_34_ carry −0.04 e. These changes in electron distribution are attributed to the coupling effect between the graphitic-N and B atoms. In Figure 2E, the reaction energy barrier of graphitic-N and B doped carbon is further reduced to 0.53 eV in step 2. Similarly, pyridinic-N and B dual doping also further disrupts the electron distribution in the carbon structure. The electron density of pyridinic-N atom increases to −0.64 e, while the electron density of B atom decreases to +0.15 e. The surrounding carbon atoms, C_12_ and C_14_, carry a charge of +0.17 e, C_23_ carries +0.03 e, while C_25_ and C_34_ carry −0.04 e. In Figure 2F, the reaction energy barrier of graphitic-N and B doped carbon is further reduced to 0.51 eV in step 2. According to the DFT calculation results, the graphitic-N, pyridinic-N and B dual-doped carbon structure further disrupts the electron cloud distribution, adjusts the adsorption energies of intermediate species O*, OH*, and OOH*, thereby lowering the energy barrier for the ORR, which is beneficial for enhancing ORR catalytic performance.

### 3.2 Physical characterization

DFT findings suggest that N, B dual-doping carbon may exhibit exceptional ORR performance. N, B dual-doping carbon ORR catalysts are synthesized from saccharina japonica.

**FIGURE 3:**
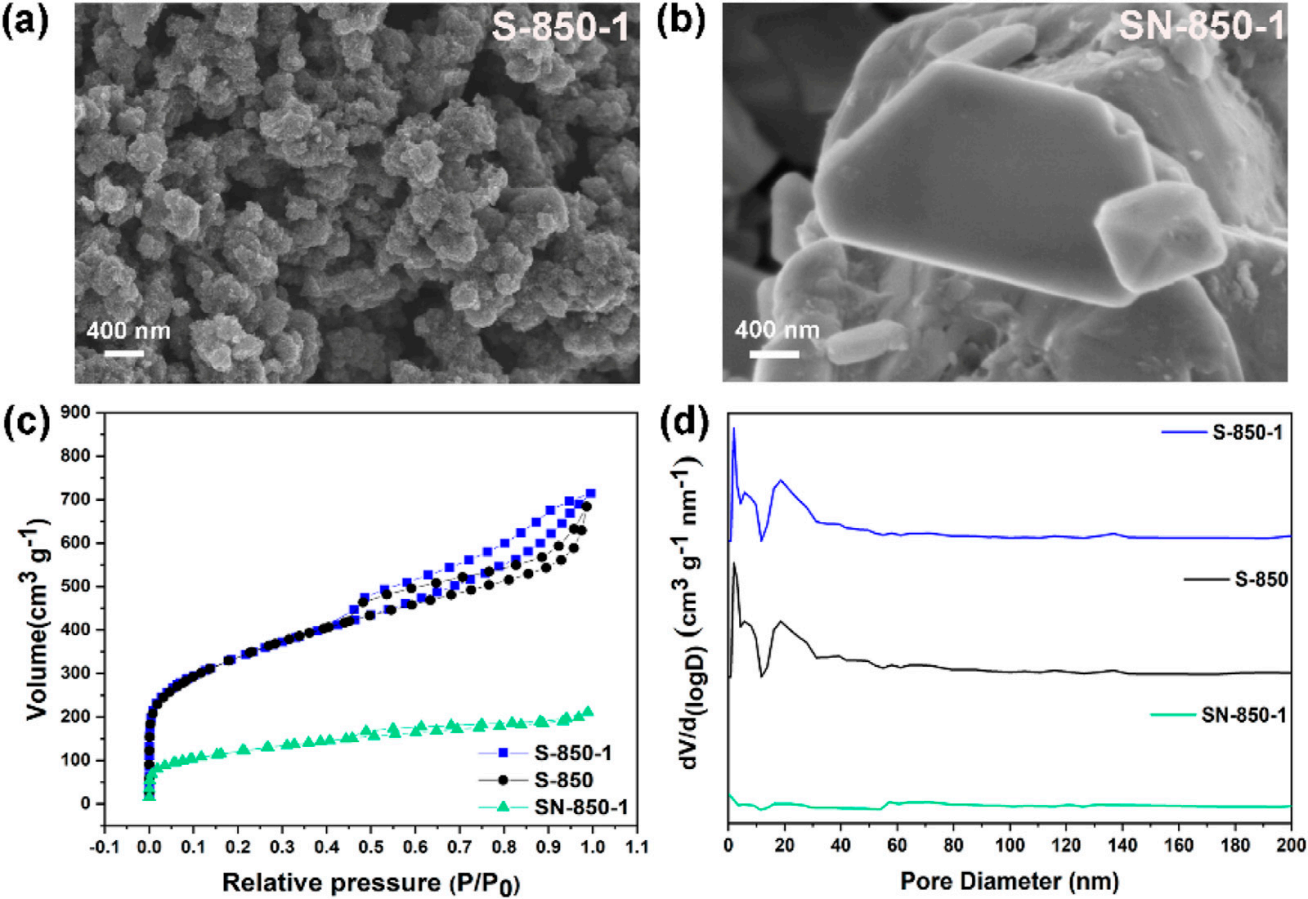
**(A, B)** SEM images of S-850-1 and SN-850-1, **(C, D)** N_2_ adsorption-desorption isotherms and pore size of the as-synthesized samples.

SEM pictures provide visual information on the shape and structure of the as-synthesized samples. In Figure 3A, S-850-1 exhibits a porous surface morphology, with interconnected carbon particles forming the surface porosity. The porous morphology enhances the exposure of active sites and improves O_2_ molecule transport. As shown in Figure 3B, SN-850-1 demonstrates a large, solid bulk structure at the micrometer scale, with a non-porous surface. SEM analysis indicates that the porous structure of the sample is attributed to the influence of ZnCl_2_ activator. N₂ adsorption-desorption study assesses the surface area and distribution of pore sizes. In Figure 3C, mesopores are evident, with both S-850 and S-850-1 exhibiting type-IV isotherms and a distinct hysteresis loop in the medium and high-pressure regions (Wang et al., 2011). However, SN-850-1 exhibits a type II isotherm, indicating a non-porous structure (Li et al., 2019). After doping with B, S-850-1 shows a modest increase in specific surface area from 1,108 to 1,173 m^2^ g⁻^1^, which is likely attributable to the B atomic incorporation. SN-850-1 exhibits a comparatively low specific surface area of 89 m^2^ g⁻^1^. Non-local density functional theory, the distribution of pore size shown in Figure 3D is predominantly in the 15–50 nm range, indicating the presence of mesopores. SN-850-1 is characterized by a non-porous structure. Based on the above physical characteristics, ZnCl_2_ activator is identified as a key factor in the development of the porous structure of biomass-derived carbon-based ORR catalyst, potentially impacting ORR performance.

**FIGURE 4:**
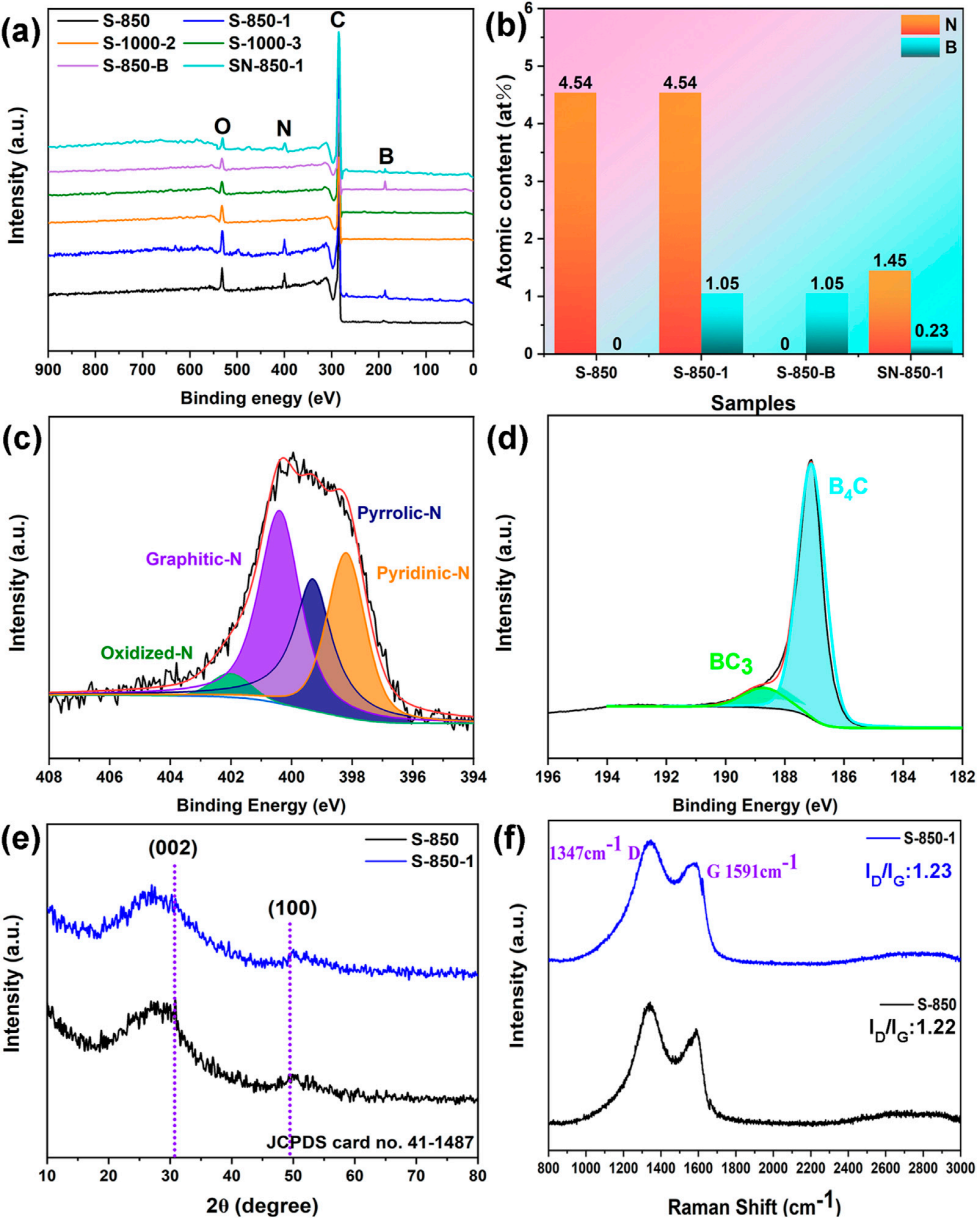
**(A)** Full-scan XPS, **(B)** Total N and B content, **(C, D)** High-resolution N and B atoms of S-850-1, **(E)** XRD pattern, **(F)** Raman spectrum.

XPS measurements can be used to analyze the types and quantities of doping N and B atoms. Figure 4A reveals the presence of doped N atoms through peaks at approximately 187 eV (B), 284 eV (C), 532 eV (O), and 400 eV (N) for the as-synthesized samples (Lee et al., 2016). S-850 contains only doped C, O, and N atoms, whereas S-850-1 exhibits significant B doping, confirming the incorporation of B atoms into the N-doped carbon matrix. S-850-1 shows a higher O content than N and B. The pyrolysis temperature of the samples is increased to 1,000°C and maintained for 2 h to ensure the complete volatilization of doped N atom. S-1000-2 is mixed with NaBH₄ in a 1:1 ratio, with the pyrolysis temperature set at 850°C and held for 2 h, resulting in the sample being named S-850-B.

Pyrolysis at 1,000°C, with hold times of 2 and 3 h (noted as S-1000-2 and S-1000-3), is conducted to fully volatilize N and B, aiming to investigate the effect of doped O atom on ORR performance. In the S-1000-2 and S-1000-3 samples, N and B are indeed absent, with only O remaining, which is attributed to the volatilization of N and B at high temperatures. The O contents of S-1000-2 and S-1000-3 are 5.34 at% and 3.21 at%, respectively. Subsequent electrochemical performance tests will be conducted to analyze the effect of doped O atom on ORR performance. N (1.45 at%) and B (0.23 at%) contents in SN-850-1 are lower than those in S-850-1 (N: 4.54 at% and B: 1.05 at%), indicating that the porous structure facilitates the exposure of more heteroatoms. Figure 4B shows doping N and B atomic content of the as-synthesized samples. The high-resolution N_1s_ spectra of S-850-1 reveal peaks corresponding to graphitic-N (401 eV), pyridinic-N (398 eV), pyrrolic-N (399 eV), and oxidized-N (402 eV) (Zhang et al., 2023b; Huang et al., 2019). The doped B atoms are represented by peaks at 187 eV (B₄C) and 189 eV (BC₃) in Figures 4C, D. According to the XPS results, saccharina japonica may serve as a precursor for the synthesis of N mono-doping carbon materials. Additionally, combining saccharina japonica with NaBH₄ enables the effective production of N and B dual-doping carbon, which may show promising activity for ORR.

XRD patterns of S-850-1 and S-850 exhibit two distinct peaks at 2θ = 30.7° and 49.4°, which match to the (002) and (100) crystallographic planes of a standard hexagonal carbon structure (JCPDS card no. 41–1,487) in Figure 4E (Yu et al., 2014). The diffraction peaks of the (002) plane in S-850 and S-850-1 show a slight negative shift relative to the standard peak, indicating that the diffraction is performed by ions (N, and B) of different radius or the defects formed due to incorporation of N or B. Both S-850 and S-850-1 display D and G bands in Raman spectra (Figure 4F). The extent of disorder in the carbon material is reflected in the I_D_/I_G_ ratio of these bands (Kim et al., 2011). For S-850-1, the I_D_/I_G_ ratio is 1.23, showing a slight increase compared to the N mono-doped S-850, which has an I_D_/I_G_ ratio of 1.22. This increases likely results from the enhanced defect density due to B doping.

### 3.3 Electrochemical characterization

**FIGURE 5:**
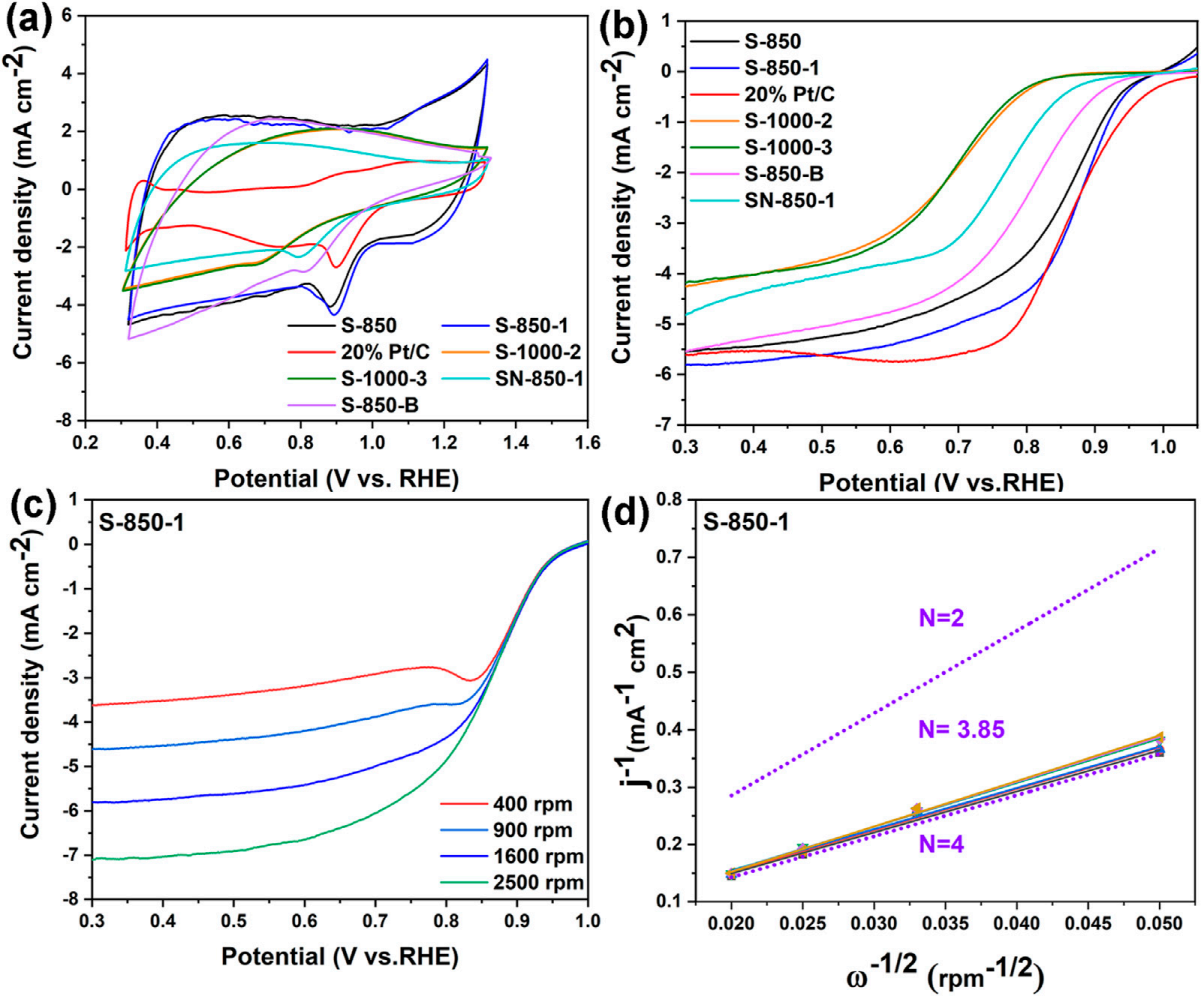
**(A, B)** CV, LSV curves of the as-synthesized samples, **(C)** RDE curves, **(D)** K-L plots of S-850-1.

This work assessed the ORR performance of the as-synthesized samples and 20% Pt/C using CV and LSV tests in 0.1 M KOH electrolyte, with recorded potentials plotted against the RHE scale. Figure 5A shows clear reduction peaks for the above samples, demonstrating their ORR activity in an alkaline electrolyte. Compared to S-850 (0.881 V) and S-850-B (0.805 V), S-850-1 exhibits a higher peak potential (0.895 V), closely matching that of 20% Pt/C (0.899 V). The results show that the N, B dual-doped carbon catalyst has significantly higher ORR performance than the N or B mono doped carbon catalyst, even approaching that of 20% Pt/C. The peak potentials of the N and B mono doped carbon catalysts (S-850 and S-850-B) are higher than those of the N, B dual-doped SN-850-1 (0.795 V) with low surface area and non-porous structure, indicating a direct relationship between surface area, porosity, and ORR performance.

Among the carbon catalysts with different doped O contents, S-1000-2 (O: 5.34 at%) and S-1000-3 (O: 3.21 at%) without N and B doping exhibit similar peak potentials of 0.682 V, which are lower than those of the N- and B-doped carbon catalysts, indicating that O doping has a minimal effect on ORR performance. Furthermore, the peak potential of the most effective S-850-1 exceeds that of previously reported N and B dual-doped carbon catalysts (BN-GNRs: 0.870 V (Tu et al., 2022), B-NHMC: 0.881 V (Al-Enizi, 2022), BNC-600: 0.759 V (Zhou et al., 2021)), which can be attributed to its lower N and B content, as well as its smaller surface area and pore structure. The LSV curves, shown in Figure 5B, demonstrate that the performance of S-850-1 is like that of 20% Pt/C. Specifically, S-850-1 shows improvements of 23 mV and 0.36 mA cm⁻^2^ in half-wave potential and limited current density (0.861 V and −5.60 mA cm^−2^), respectively, compared to S-850 (0.838 V and −5.24 mA cm^−2^). The half-wave potential and limited current density of S-850-1 are nearly identical to those of 20% Pt/C. While S-850-1 starts with an initial potential of 0.962 V, it shifts negatively by 83 mV compared to the 1.050 V of 20% Pt/C. The trends observed in the onset potential, half-wave potential, and limited diffusion current density of the other samples are consistent with the above CV results. The detailed electrochemical data are listed in Table 1.

**TABLE 1:** Electrochemical characterizations of the as-synthesized samples and previously reported N, B-doped carbon catalysts.

Samples	Peak potential (V)	Onset potential (V)	Half-wave potential (V)	Limited current density @0.5V (mA cm^-2^)
S-850	0.881	0.912	0.838	−5.24
S-850-1	0.895	0.962	0.861	−5.60
20% Pt/C	0.899	1.050	0.862	−5.60
S-850-B	0.805	0.976	0.785	−5.04
SN-850-1	0.795	0.935	0.768	−4.10
S-1000-2	0.682	0.890	0.640	−3.76
S-1000-3	0.682	0.890	0.641	−3.78
BN-GNRs (Tu et al., 2022)	0.870	0.954	0.852	−4.00
B-NHMC (Al-Enizi, 2022)	0.881	0.975	0.835	−4.20
BNC-600 (Zhou et al., 2021)	0.759	0.930	0.790	−5.10

Based on the preceding physical and electrochemical test results, the N, B dual-doped carbon structure demonstrates a notable enhancement in ORR performance, surpassing that of the mono N and B doped carbon structures. Furthermore, the ORR activity of the N, B dual-doped carbon catalyst S-850-1 exceeds that of previously reported N, B dual-doped materials in the literature, suggesting that S-850-1 holds substantial potential for commercialization as an ORR catalyst. The LSV curves (rotation rates: 400–2,500 rpm), shown in Figure 5C, demonstrate that higher rotation rates increase the limiting diffusion current density, reduce diffusion distance, and enhance oxygen transport (Tan et al., 2012). K-L plots within the 0.3–0.7 V potential range reveal strong linearity for S-850-1, suggesting first-order reaction kinetics for oxygen reduction (Xu et al., 2017). Moreover, S-850-1 has an electron transfer number (n) of 3.85, indicating a predominant 4-electron ORR way.

**FIGURE 6:**
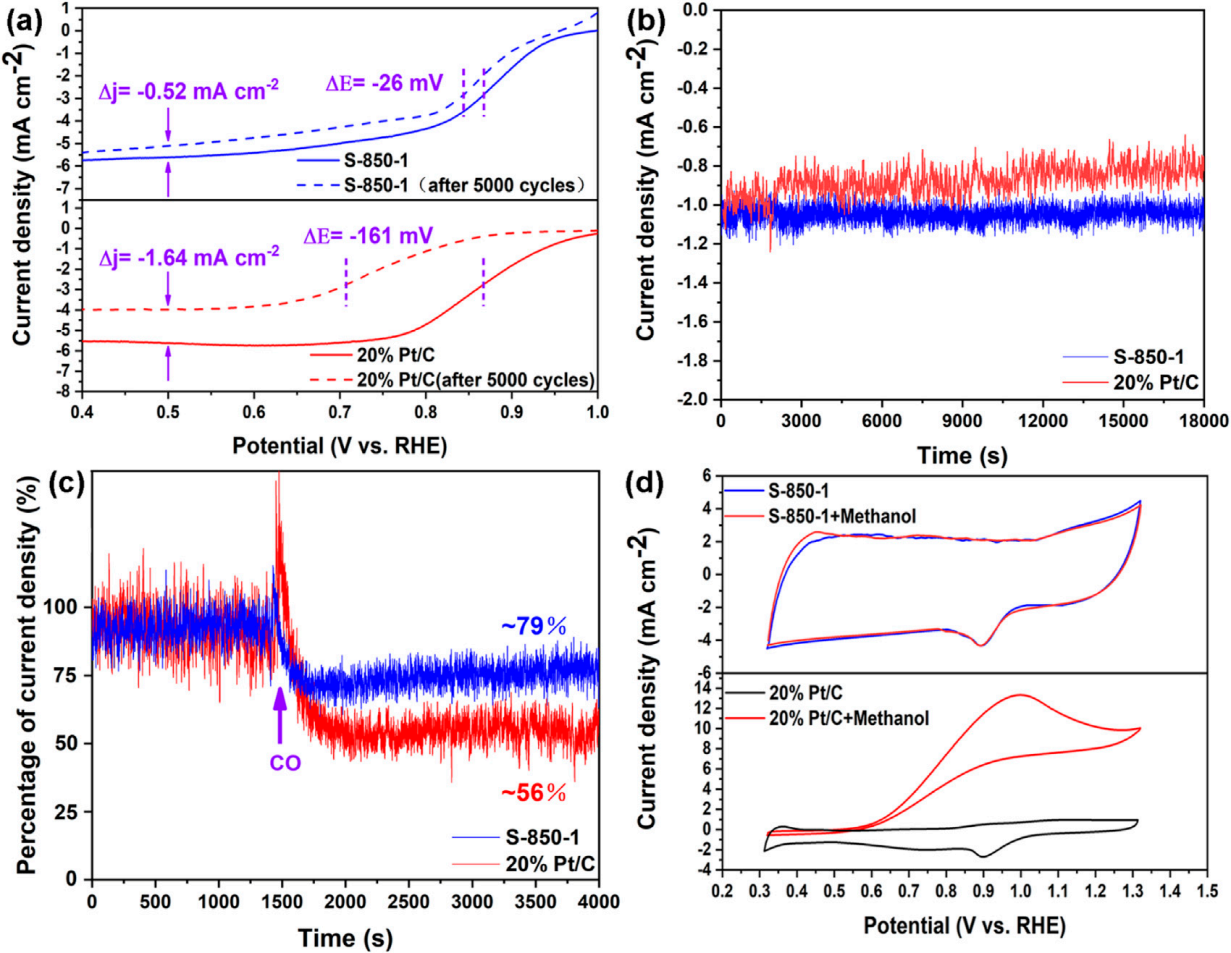
**(A)** LSV curves pre- and post-5000 CV cycles, **(B)** Chronoamperometric measurements, **(C)** Chronoamperometric measurements are obtained, with the inflection point indicating the introduction of CO, **(D)** CV curves in solution and in the presence of methanol.

The longevity of catalysts is a key challenge for fuel cell commercialization (Wu et al., 2011). After 5,000 CV cycles, S-850-1 shows a negative shift of 26 mV in half-wave potential (@1,600 rpm) and a decrease of −0.52 mA cm⁻^2^ in limiting diffusion current density from its initial values. In comparison, commercial 20% Pt/C experiences a more significant decline, with shifts of 161 mV and −1.64 mA cm⁻^2^, respectively (Figure 6A). The outstanding stability of S-850-1 may be ascribed to the robust covalent C-N bonds and the lack of complications such as Pt nanoparticle aggregation and dissolution (Zhong et al., 2013). Additionally, under chronoamperometric testing at 0.5 V for 18,000 s, the current density of 20% Pt/C drops to 0.8 mA cm⁻^2^, while S-850-1 maintains a current density of approximately 1.1 mA cm⁻^2^ (Figure 6B). CO gas poses a significant challenge for fuel cells by increasing the risk of ORR catalyst poisoning. Figure 6C illustrates the catalysts’ tolerance to CO. Upon CO introduction at 4,000 s, 20% Pt/C’s current density decreases to about 56%, indicating a higher susceptibility to CO poisoning compared to S-850-1, which retains about 79% of its current density. In direct methanol fuel cells, methanol may migrate from the anode to the cathode, where it can potentially poison the ORR catalyst (Gao et al., 2015c). Therefore, methanol tolerance is crucial for optimal catalyst performance. As shown in Figure 6D, S-850-1 demonstrates high selectivity for oxygen and shows no significant activity loss upon the addition of 10 mL methanol, indicating effective resistance to methanol crossover. In contrast, the ORR performance of 20% Pt/C is adversely affected by methanol, as it is more prone to methanol oxidation reactions (MOR). These results highlight that S-850-1 surpasses 20% Pt/C in terms of stability and resistance to methanol and CO poisoning.

**FIGURE 7:**
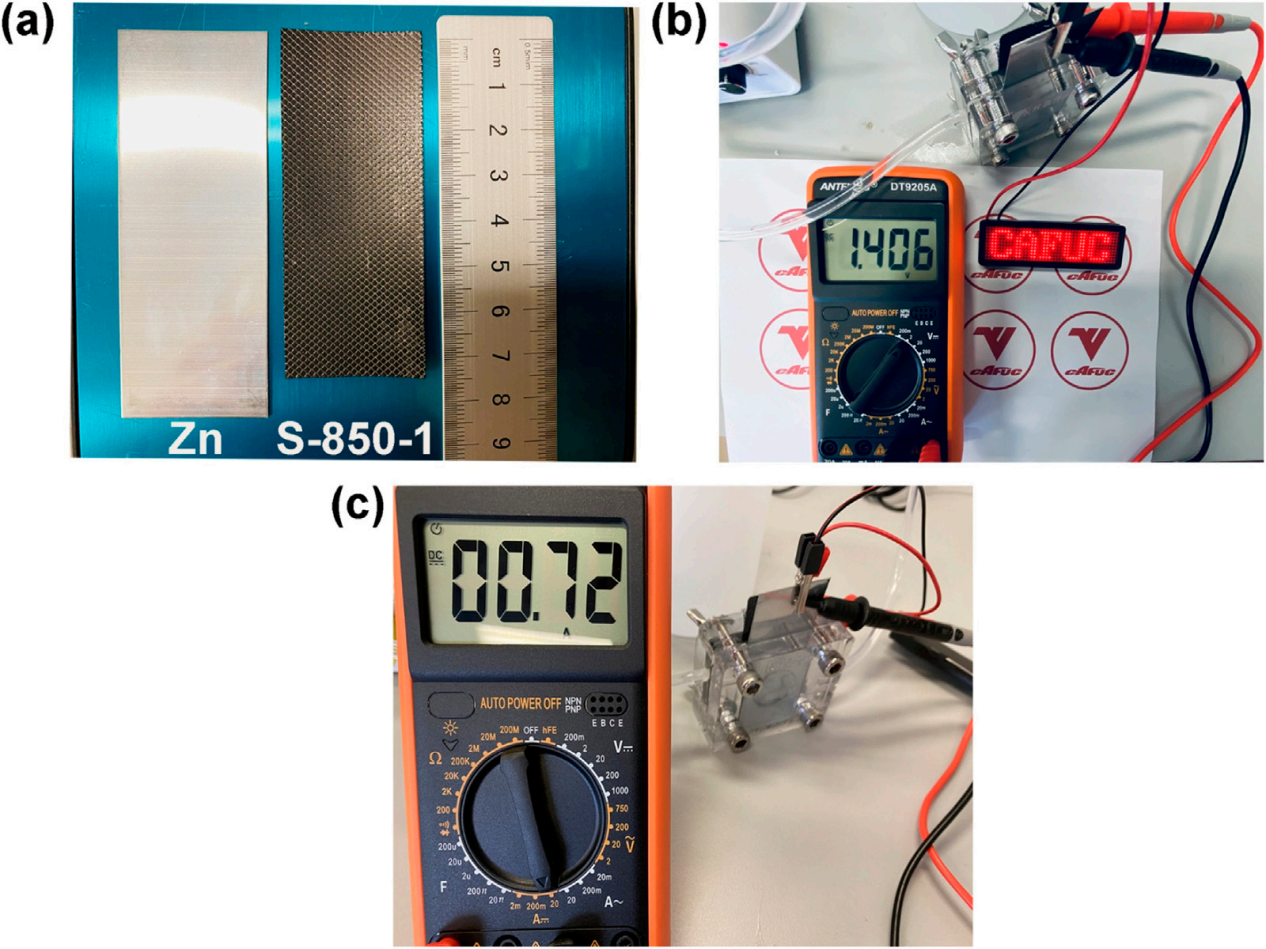
**(A)** S-850-1 and Zn plate, **(B)** Open circuit voltage, **(C)** Current density of Zn-air battery.

This study constructs a Zn-air battery to further investigate the performance of S-850-1. The Zn-air battery features a 4.5 cm^2^ operating surface, with a 6 M KOH solution circulating at 20 mL per minute as the electrolyte. The oxide layer is removed by sanding the zinc plate (length: about 8.5 cm, width: about 3 cm), as shown in Figure 7A. S-850-1 coated on stainless steel mesh (approximate length: 7.5 cm, approximate width: 3 cm). The Zn-air battery’s open circuit voltage and current density in Figure 7B, c are 1.40 V and 0.72 A, respectively, which illuminates the LED light. The findings suggest that S-850-1 may be used as an ORR catalyst for Zn-air batteries.

Future research directions will focus on exploring novel biomass that is inherently rich in various heteroatoms, enabling the formation of self-doped carbon-based ORR catalysts with high heteroatom content. This approach will avoid the use of external doping chemicals and reduce preparation costs. In the process of scaling up the industrial production of biomass-derived carbon-based ORR catalysts, challenges may arise due to variations in the heteroatom content caused by differences in the origin and seasonal changes of biomass materials across different batches. Additionally, it is crucial to develop scalable and cost-effective processes, such as ball milling, to produce heteroatom-doped carbon-based electrocatalysts. These processes should ensure the catalysts possess optimal structural and surface chemical properties to meet the demands of industrial applications (Sekhon et al., 2022; Koolen et al., 2023).

## 4 Conclusion

The study successfully develops an efficient N, B dual-doped carbon ORR catalyst (S-850-1) using Saccharina japonica, DFT calculations, NaBH₄ doping, and pyrolysis methods. Initially, six theoretical models demonstrate that N and B dual-doping carbon offers superior ORR performance compared to N and B mono-doped carbon. Saccharina japonica and NaBH₄ serve as the precursor and boron source, respectively, for pyrolyzing N (4.54 at%) and B (1.05 at%) dual-doped carbon (S-850-1) at 850°C. The ORR activity of S-850-1 outperforms that of N mono-doped carbon (S-850), evidenced by its higher half-wave potential (0.861 vs. 0.838 V) and greater limited current density (−5.60 vs. −5.24 mA cm⁻^2^). This indicates that N, B dual-doping carbon has enhanced ORR performance. Furthermore, S-850-1 is utilized to fabricate a Zn-air battery with an open circuit voltage of 1.40 V, successfully powering an LED light. These electrochemical results corroborate the DFT predictions, suggesting that S-850-1, derived from the widely available Saccharina japonica, has the potential to be a commercially viable ORR catalyst for the next generation of fuel cells.

## Data Availability

The original contributions presented in the study are included in the article/supplementary material, further inquiries can be directed to the corresponding authors.
